# Large genomic rearrangements in BRCA1 and BRCA2 genes in breast and ovarian cancer families in Poland

**DOI:** 10.1186/1897-4287-10-S3-A2

**Published:** 2012-04-20

**Authors:** Helena Bielecka, Bohdan Górski

**Affiliations:** 1 Pomeranian Medical University, Clinic of Oncology, Szczecin, Poland

## 

Mutations in the BRCA1 and BRCA2 genes predispose women to breast and ovarian cancer. The large majority of the alterations identified in these genes are point mutations and small insertion/deletion. However, an increasing number of large genomic rearrangements (LGRs) are being identified, especially in BRCA1. To date just a few large genomic rearrangements of BRCA1 gene have been reported in Poland. Technical limitations of conventional PCR-based methods are cause that gross rearrangements can be overlooked. It has been suggested that about 30% of mutations in the BRCA1 gene are missed by standard mutation detection methods. We screened for LGRs in BRCA1 and BRCA2 genes by Multiplex Ligation-dependent Probe Amplification (MLPA) in 200 unrelated patients with strong family history of breast and/or ovarian cancer negative for BRCA1 Polish founder mutation. We identified 3 different LGRs in BRCA1 gene. No large LGRs were detected in BRCA2 genes.

